# Transmembrane Helices Are an Overlooked Source of Major Histocompatibility Complex Class I Epitopes

**DOI:** 10.3389/fimmu.2017.01118

**Published:** 2017-09-11

**Authors:** Frans Bianchi, Johannes Textor, Geert van den Bogaart

**Affiliations:** ^1^Department of Tumor Immunology, Radboud Institute for Molecular Life Sciences, Radboud University Medical Center, Nijmegen, Netherlands

**Keywords:** antigen presentation, antigen cross-presentation, membrane proteins, bioinformatics, adaptive immunity, transmembrane domain, epitopes, T lymphocyte

## Abstract

About a fourth of the human proteome is anchored by transmembrane helices (TMHs) to lipid membranes. TMHs require multiple hydrophobic residues for spanning membranes, and this shows a striking resemblance with the requirements for peptide binding to major histocompatibility complex (MHC) class I. It, therefore, comes as no surprise that bioinformatics analysis predicts an over-representation of TMHs among strong MHC class I (MHC-I) binders. Published peptide elution studies confirm that TMHs are indeed presented by MHC-I. This raises the question how membrane proteins are processed for MHC-I (cross-)presentation, with current research focusing on soluble antigens. The presentation of membrane-buried peptides is likely important in health and disease, as TMHs are considerably conserved and their presentation might prevent escape mutations by pathogens. Therefore, it could contribute to the disease correlations described for many human leukocyte antigen haplotypes.

Human leukocyte antigen (HLA) molecules play a vital role in the immunological T cell response. HLA-A and HLA-B code for major histocompatibility complex (MHC) class I, which mainly presents peptide fragments derived by proteasomal degradation of “self” proteins on the cell surface of the antigen presenting cell to cytolytic T cells (1–3). HLA encoding genes are highly polymorphic and over 8,500 unique HLA-A and HLA-B haplotypes have been identified to date (4, 5), each presenting different peptide fragments of mostly nine amino acids in length (6). This polymorphism provides an evolutionary advantage, because pathogens cannot easily develop resistance by mutating residues critical for binding to all HLA haplotypes. In this article, we reason that another way how resistance is prevented is by the presentation of stretches of amino acids which are critical for protein function: those located within transmembrane helices (TMHs) of integral membrane proteins.

Each HLA-A and HLA-B haplotype presents peptides with preferential requirements for the charge and hydrophobicity of each residue within the peptide (7). Based on these requirements for HLA binding, the haplotypes of HLA-A and HLA-B can be grouped in five and seven super types, respectively (7–10). The binding peptides (binders) for these super types can be predicted from protein sequences by bioinformatics tools (11, 12), whose high accuracy was empirically confirmed in several studies (13–16). For most HLA-A and HLA-B super types, the presence of multiple hydrophobic residues is favorable for peptide binding (7).

The occurrence of multiple hydrophobic residues in close proximity of each other is a hallmark of TMHs. Due to the hydrophobic nature of lipid membranes, a transmembrane motif of mostly 23 amino acids in length containing non-polar headgroups is required to span the membrane (17). These stretches are frequently flanked by bulky hydrophobic residues, such as tryptophan, that allow for anchoring of the protein at the water–lipid interface, and by positively charged lysine and arginine residues that allow for electrostatic interactions with negatively charged lipid headgroups (18–20). Similar to HLA binders, the presence of TMHs can be predicted with high accuracy from protein sequences (21). Approximately 25% of all human genes code for integral membrane proteins that are anchored by one or more TMHs to the plasma membrane or to the lipid membranes of organelles.

Since both HLA-A/B binders and TMHs contain multiple hydrophobic residues, it is likely that HLA-A and HLA-B haplotypes would preferentially present peptides derived from TMHs. This is supported by a comparison of the predicted binders with predicted TMHs from the human proteome (Figure 1A). Predicted TMHs contain more predicted HLA binders than expected from a random distribution of binders for all HLA-A and most HLA-B super types, and this was significant for all HLA super types (see Methods; *P* < 0.001; individual *P* values in Table S1 in Supplementary Material). Comparing the hydrophobicity (22) of all peptides in the human proteome reveals that peptides derived from TMHs are more hydrophobic than those originating from other protein regions (Figure 1B) and more than 90% of the 10% most hydrophobic peptides originate from TMHs (Figure 1C).

**Figure 1:**
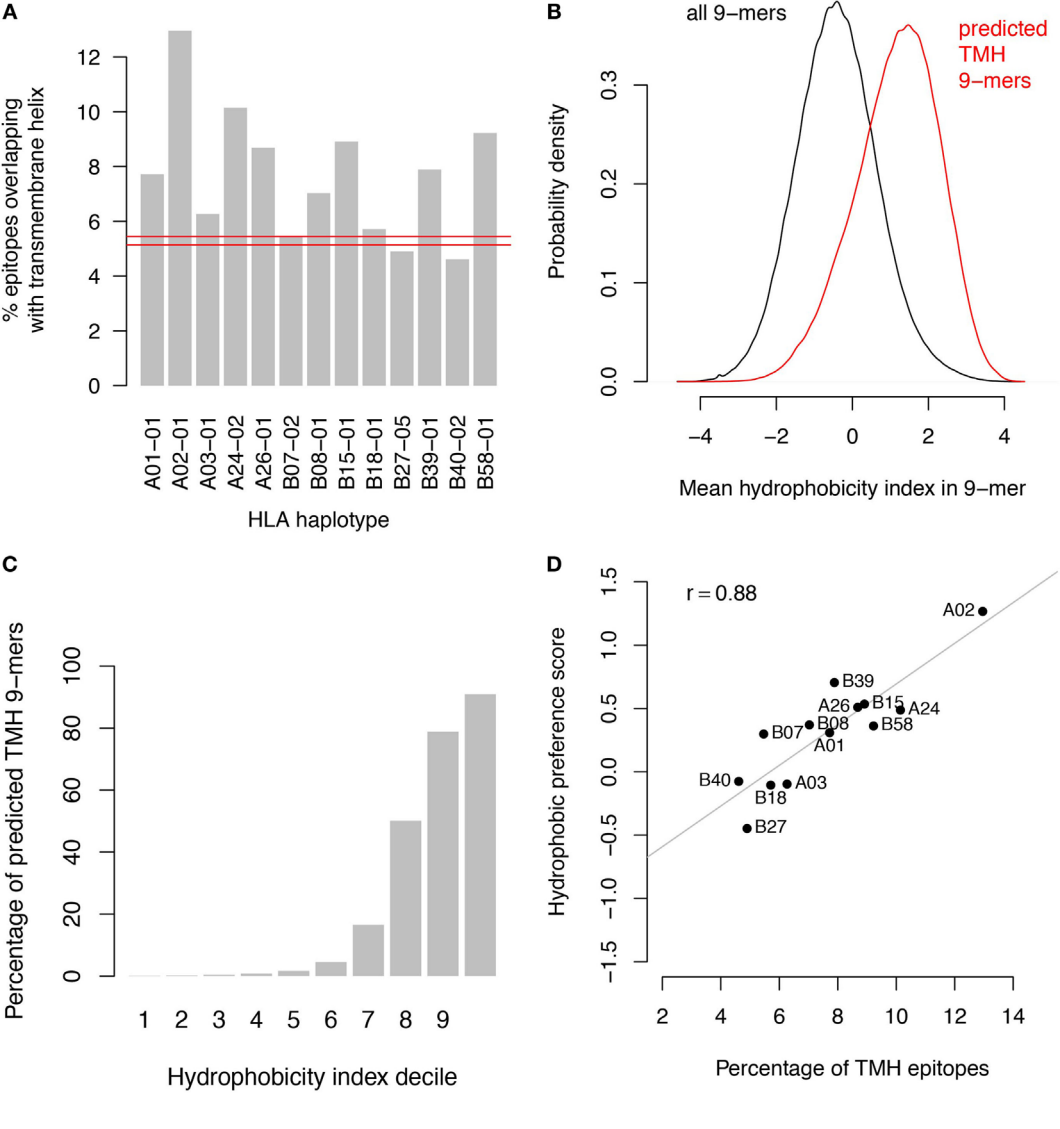
Transmembrane-derived peptides are preferentially bound by major histocompatibility complex (MHC) class I haplotypes due to their hydrophobic nature. **(A)** Epitopes derived from transmembrane helices (TMHs) are over-presented by all human leukocyte antigen (HLA)-A and most HLA-B super types. The bar graphs show the percentages of predicted binders for all HLA-A and HLA-B super types from the human proteome overlapping with predicted TMHs by at least one residue. Predictions of MHC class I-binding peptides of nine amino acids in length from the human proteome were made using the stabilized matrix method. These were compared with all TMHs predicted by TMHMM version 2.0 (23). The red lines show the 99.9% confidence interval of overlapping binders based on random distribution over the proteome. **(B)** Hydrophobicity distribution of all peptides in the human proteome (black curve) compared to peptides overlapping with TMHs (red curve). **(C)** Percentage of peptides overlapping with TMHs per decile of hydrophobicity. **(D)** Percentage of TMH-overlapping predicted binders versus hydrophobic preference score for each individual haplotype.

We performed two control analyses to determine to what extent the preferential HLA binding of TMH-derived peptides was related to their hydrophobicity. First, we scored the hydrophobic preference for each HLA super type (see Methods). Haplotypes for which this score is positive will bind peptides with above average hydrophobicity. We found a strong correlation (*r* = 0.88) between these hydrophobic preference scores and the percentages of predicted HLA-binding peptides derived from TMHs (Figure 1D). For a second control, we selected a set of peptides derived from non-transmembrane protein regions of the human proteome, but with a matching hydrophobicity distribution as of those derived from TMHs (Figure 2A). If the predicted preferential presentation of peptides locating to TMHs were entirely attributable to their large hydrophobicity, we would expect these control peptides to be equally well presented. Indeed, this was largely the case (Figure 2B), except for HLA-A02. For HLA-A02, peptides derived from TMHs were predicted to be presented with a lower probability than peptides from the control set. Possibly this may be due to the specific compositional requirements of TMHs resulting in less-favorable positioning of residues as in other hydrophobic domains critical for HLA-A02 binding.

**Figure 2:**
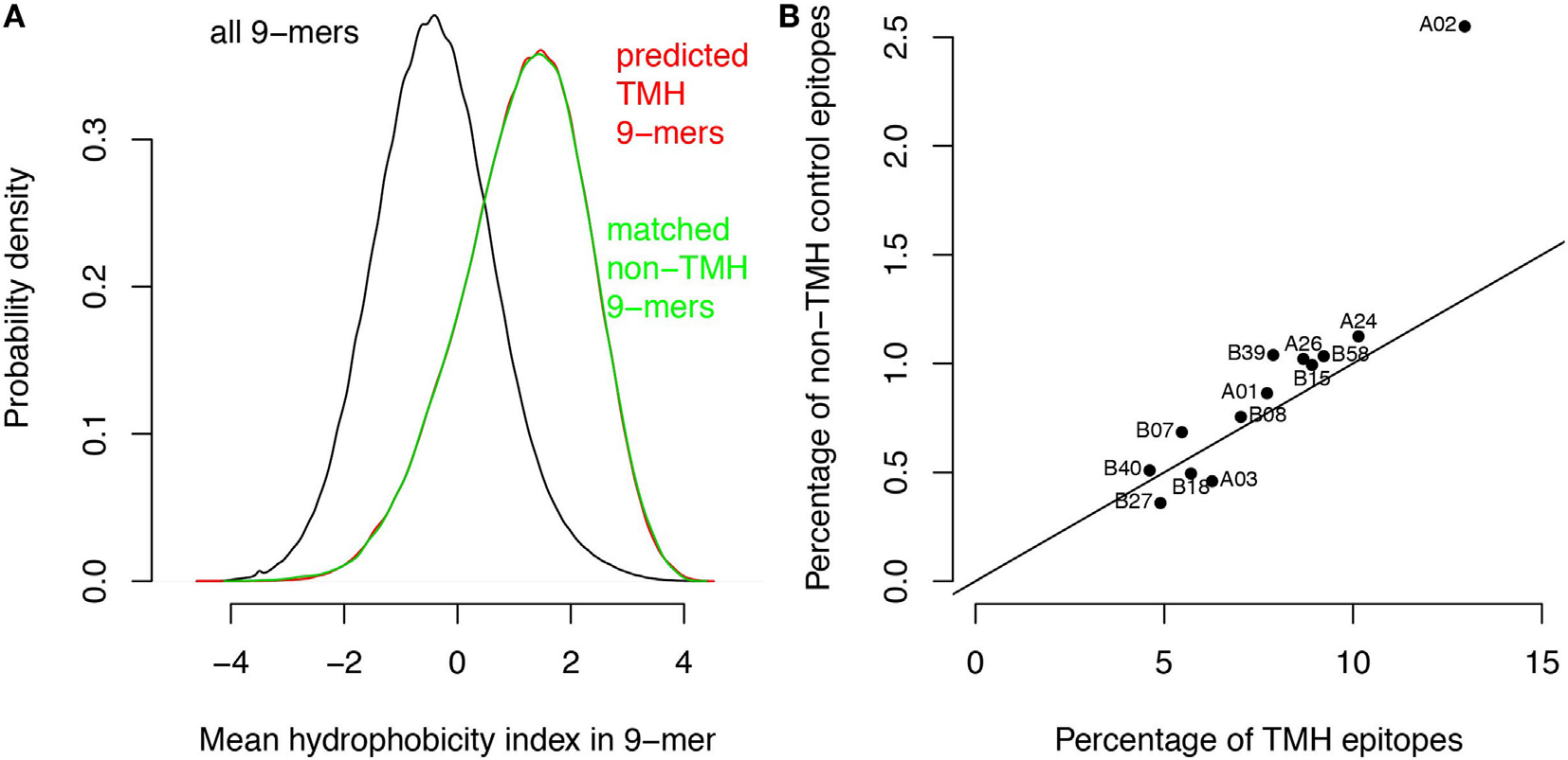
The major histocompatibility complex class I binding preference of transmembrane helix (TMH)-derived peptides is mainly attributable to their large hydrophobicity. Based on the hydrophobicity distribution of the peptides overlapping with TMHs, a control set of peptides was generated with a similar hydrophobicity distribution but now derived from soluble protein regions (i.e., non-TMHs). **(A)** Predicted hydrophobicity distribution of all peptides in the human proteome (black curve), peptides overlapping with TMHs (red curve), and the control set from soluble protein regions (green curve). Note the almost complete overlap of the red and green curves. **(B)** Percentages of predicted binders from the control set of soluble protein regions *versus* the percentage of predicted binders from TMHs (Figure 1A) for each human leukocyte antigen (HLA)-A and HLA-B super type. The black line shows the expected relationship if there were no differences between the two sets except their sizes (note that the control set is only 1/10 of the number of peptides derived from TMHs, resulting in lower percentages; see Methods).

To investigate this compositional bias further, we also determined the positions of the predicted binders relative to the positions of the predicted TMHs (Figure 3). These positions seem to correlate well with the requirements for peptide binding to the different HLA haplotypes. For instance, predicted binders of the HLA-A02 super type are mainly located within the hydrophobic core of TMHs, which is consistent with the requirement of multiple hydrophobic amino acids within the peptides (7). In contrast, binders for the HLA-B27 super type are enriched at the lipid–water interface of TMHs, correlating to its preference for charged amino acids within the epitope (7).

**Figure 3:**
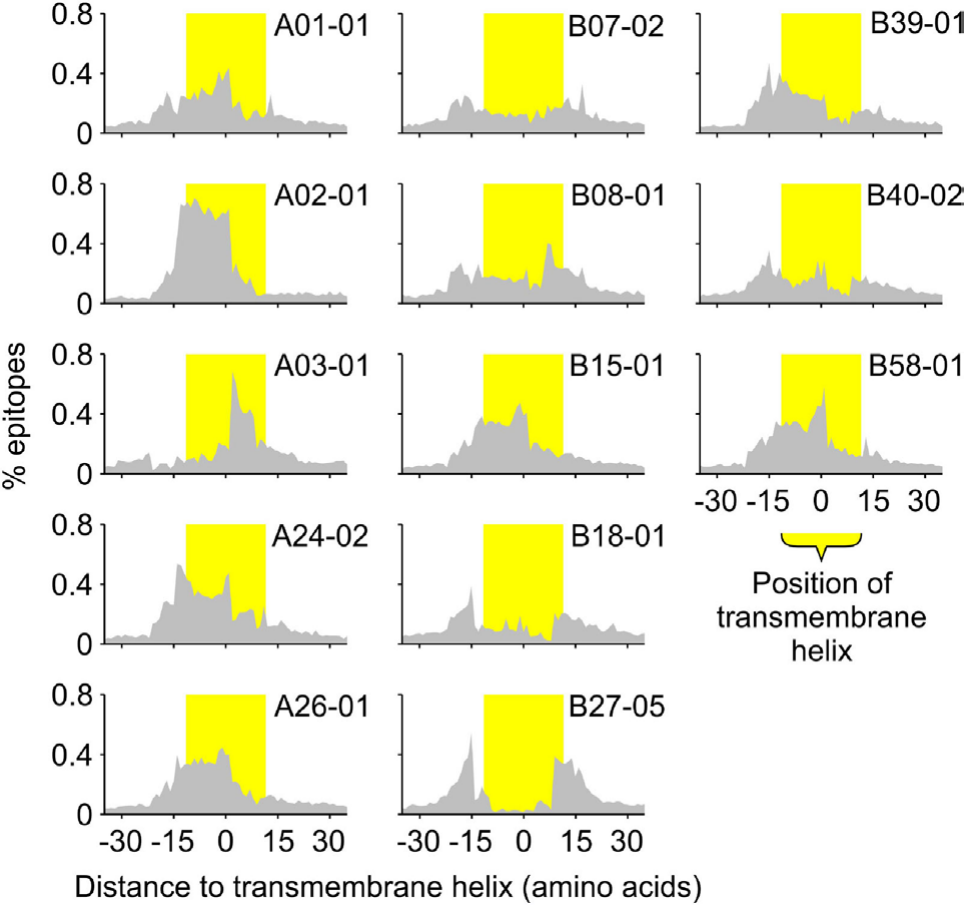
All human leukocyte antigen-A and B super types preferentially present specific sections of TMHs. The histograms show the distribution of the middle positions of all predicted major histocompatibility complex class I-binding 9-mers relative to the middle position of the nearest predicted TMH of 23 residues in length. The position of the TMH is indicated by the yellow shaded bar.

A survey of the literature confirmed that membrane-buried peptides are presented in MHC-I. Comparison of the predicted TMHs with the MHC-I epitopes identified from B lymphoblastoid cell lines by peptide elution coupled to mass spectrometry, revealed that approximately 1% of naturally presented MHC-I epitopes are predicted to be located within TMHs (24). The hydrophobicity of these detected epitopes is somewhat lower than of the predicted binders (compare Figure 4A with Figure 1B), and approximately 15% of the 10% most hydrophobic detected epitopes is derived from TMHs (Figure 4B). This percentage is lower than one would expect purely based on the predicted binding affinity (Figure 1C), which might be due to a lower abundancy of transmembrane proteins compared to soluble proteins. Alternatively or additionally, the lower percentage of peptides derived from TMHs might be caused by a lower detection efficiency, as hydrophobic peptides are often underrepresented in mass spectrometry due to their poor solubilization and ionization characteristics (25, 26). In any case, this survey of peptide elution studies (24) shows that MHC-I presents epitopes derived from TMHs.

**Figure 4:**
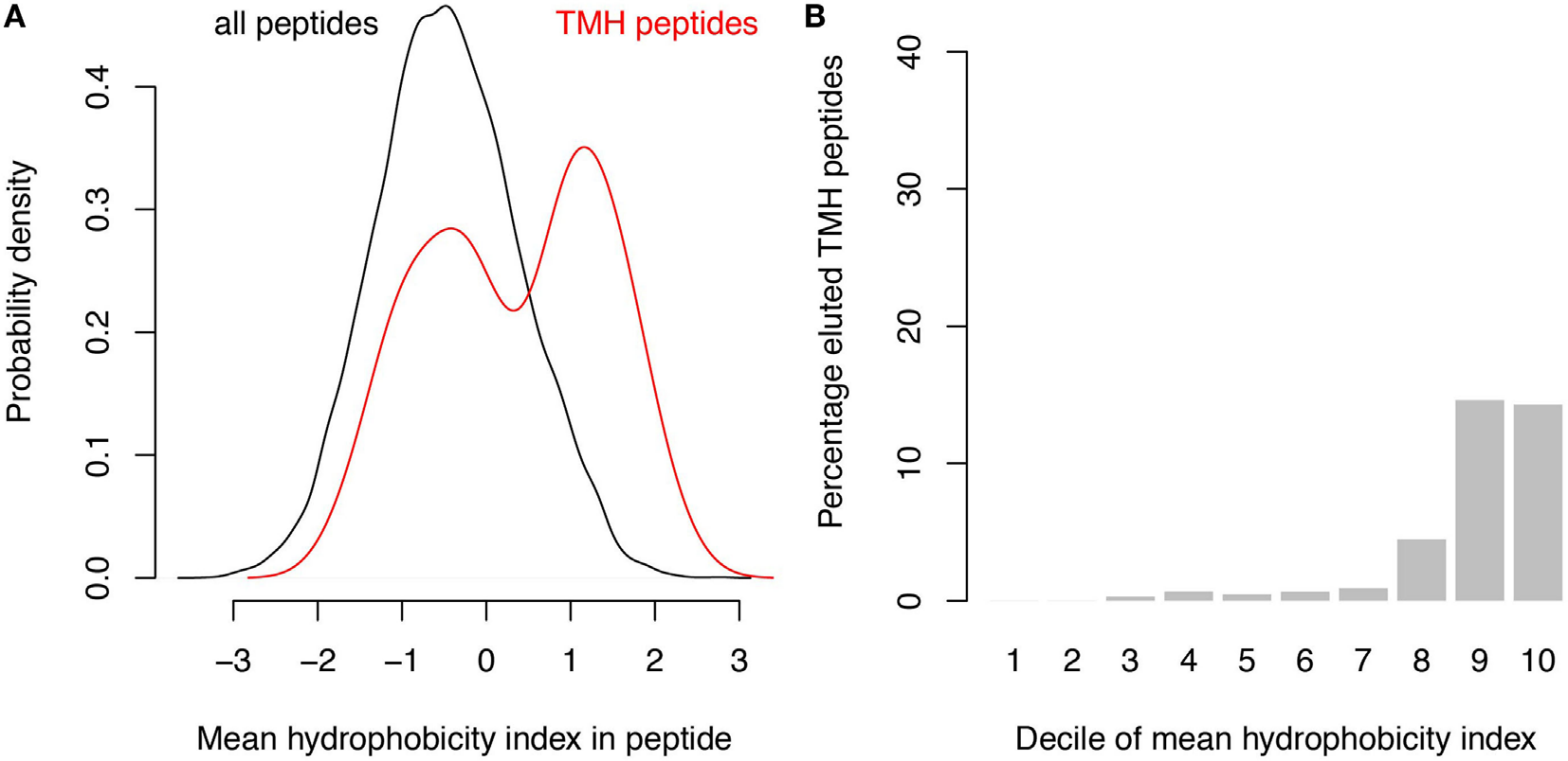
Analysis of peptide elution studies reveals major histocompatibility complex class I presentation of epitopes derived from transmembrane helices (TMHs). Peptides eluted from B lymphoblastoid cell lines and detected by mass spectrometry (24) were analyzed for their hydrophobicity and overlap with TMHs. **(A)** Hydrophobicity index distribution of all eluted epitopes (black curve) compared to epitopes derived from TMHs (red curve). **(B)** Percentage of detected epitopes derived from TMHs per decile of hydrophobicity.

In fact, many known MHC-I-binding epitopes locate within transmembrane helices. For HLA-A02, which is the most prevalent HLA-A haplotype in the Caucasian population (27), more than 10% of the epitopes in the Immune Epitope Database (28) correspond to peptide fragments overlapping with TMHs. These include many well-studied and clinically important epitopes that were found in *in vivo* studies. For example, multiple HLA-A02-binding epitopes are derived from the TMH of the viral coat protein gp41 from the human immunodeficiency virus (29). The same holds true for cancer recognition, and for instance an HLA-A02-binding epitope derived from the TMH of melanoma antigen recognized by T-cells 1 (Mart1; also known as Melan-A; residues 26–35) (30) is strikingly immunodominant, with few other MHC-I epitopes described (31).

The presentation of epitopes derived from TMHs could offer a clear evolutionary advantage. TMHs are well-conserved as there are strict compositional requirements for spanning lipid membranes (17). In addition to a hydrophobic core, the charge distribution of adjacent residues is a critical determinant for the orientation of a TMH within the membrane (32). The length of TMHs is important for the localization of transmembrane proteins to the correct organellar membrane (33). Because the precise composition of TMHs is essential for protein conformation and localization, pathogens might be unable to evade antigen presentation by mutating these regions. We believe this could constitute a novel mechanism contributing to the fidelity of antigen presentation.

It is mechanistically unclear how TMHs can be processed for MHC-I antigen presentation. For presentation of self-coded antigens in MHC-I, membrane proteins need to be first extracted from the membrane and subsequently targeted to the proteasome for the generation of antigenic epitopes (Figure 5). To date, this extraction has only been described for transmembrane proteins in the endoplasmic reticulum (ER) by a process that involves the ER-associated degradation pathway. This incompletely understood process is predominantly being studied in yeast (34, 35) and it is unclear if and to what extent it contributes to antigen presentation in mammals. However, self-presentation of an epitope derived from the TMH of Mart1 is dramatically enhanced with a mutant form that is selectively retained at the ER (36), suggesting that extraction of proteins from the ER membrane may well be of major importance for antigen presentation. Further evidence that TMH-derived epitopes are generated at the ER comes from the finding that a peptide fragment from the TMH of Mart1 is trimmed by the ER-associated proteases (ERAP) to release the MHC-I epitope (37).

**Figure 5:**
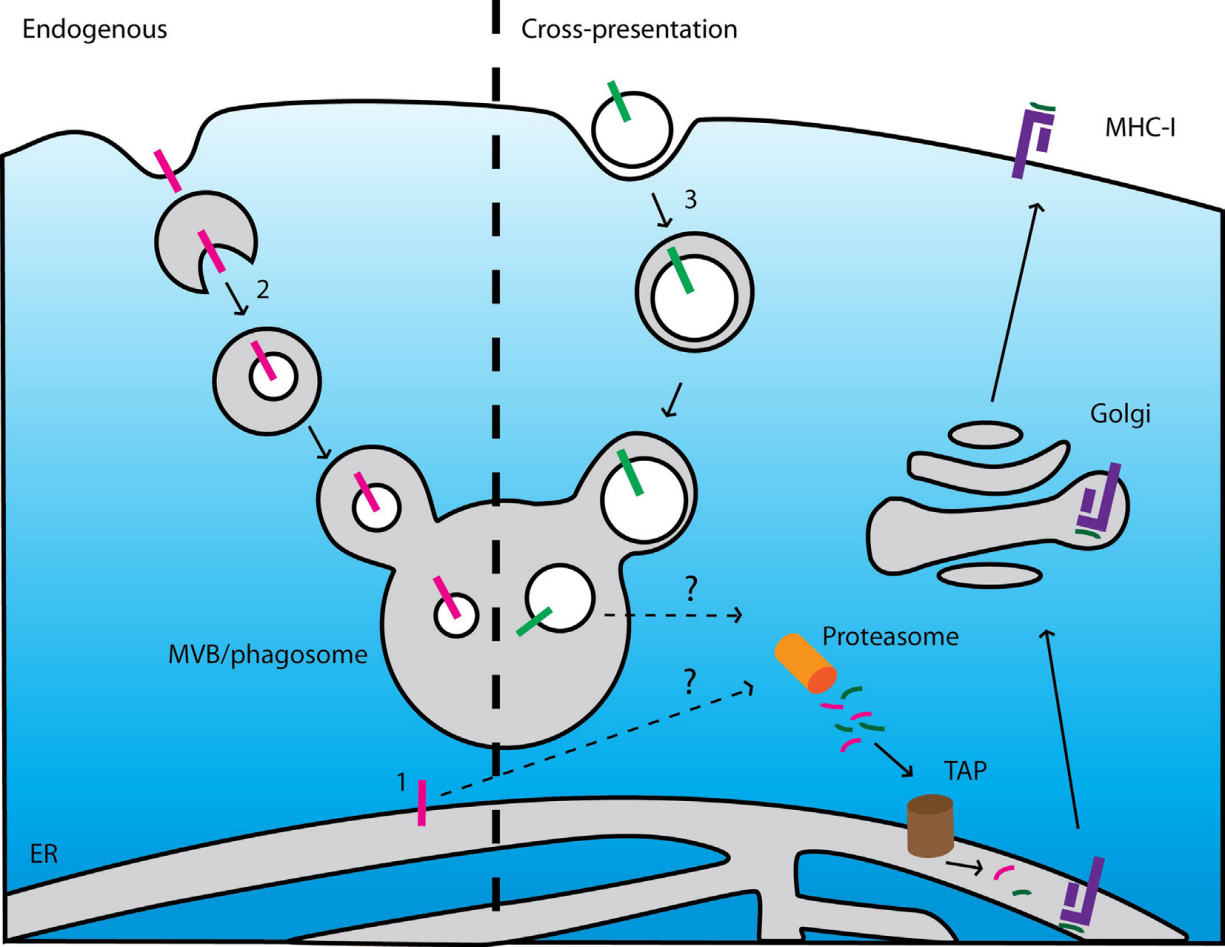
Current models cannot explain presentation of epitopes derived from endogenous and exogenous membrane proteins. Left: scheme of major histocompatibility complex (MHC) class I (MHC-I) presentation of membrane-buried epitopes derived from endogenous membrane proteins. (1) ER-resident membrane proteins can be degraded after extraction from the membrane by an ER-associated degradation-dependent mechanism and subsequently degraded by the proteasome. (2) Membrane proteins locating at other organelles can be targeted to intraluminal vesicles of multi-vesicular bodies (MVB) by the ESCRT machinery. After fusion of the MVBs with lysosomes, these vesicles (including the transmembrane proteins) are degraded by lysosomal enzymes. If and how these pathways contribute to MHC class I (MHC-I) presentation is unclear. *Right*: scheme of cross-presentation of membrane-buried epitopes derived from exogenous proteins. (3) Membrane structures (e.g., apoptotic bodies, microbial pathogens) are internalized by endocytosis or phagocytosis. The endosomes or phagosomes containing the membrane-buried epitopes fuse with lysosomes resulting in degradation of the membrane proteins. How these membrane-buried epitopes can be extracted from the membrane and loaded onto MHC-I is unknown.

The processing of TMHs for MHC-I presentation may well be mechanistically reminiscent to HLA-E presentation. HLA-E is a paralog of MHC-I specialized in the presentation of epitopes derived from the N-terminal signal peptides of other MHC-I paralogs, including HLA-A and HLA-B, for recognition by natural killer cells (38). These signal peptides resemble TMHs and are extracted from the ER membrane following cleavage by the signal peptide peptidase (39), and subsequently processed by the proteasome for HLA-E presentation (40). In this respect, it is interesting that downregulation of ERAP1 leads to upregulation of HLA-E, suggesting a functional link between HLA-E presentation and conventional antigen presentation (41).

It is also unclear whether and *via* which mechanisms transmembrane proteins locating at other organelles (i.e., outside the ER) are processed for MHC-I presentation. These transmembrane proteins are degraded in lysosomes *via* the formation of multi-vesicular bodies (Figure 5) (42). If and how these mechanisms contribute to the release of epitopes from TMHs for MHC-I presentation is yet unknown.

How integral membrane proteins can be processed for antigen cross-presentation is unclear as well. Cross-presentation is the process by which antigen presenting cells take up, process, and eventually present extracellular antigen on MHC-I to cytolytic T lymphocytes. There are currently two mechanistic pathways of antigen cross-presentation described (43), but for both pathways it is not easy to envision how they could result in cross-presentation of transmembrane proteins (Figure 5). In the first cytosolic pathway, ingested proteins translocate from endosomes into the cytosol where they become accessible for proteasomal degradation. The proteasome-derived peptides can then be imported into the ER or back into endosomes for subsequent loading onto MHC-I. The processing of transmembrane proteins would require either (i) the extraction of the TMHs from the lipid membrane within the lumen of endosomes or (ii) the translocation of large membrane fragments incorporating the antigen. In the second vacuolar pathway, proteins are degraded within endosomes by lysosomal proteases and subsequently loaded onto MHC-I. For processing of transmembrane proteins *via* this pathway, the TMHs need to be also extracted from the membrane or (iii) cleaved within the lipid membrane. All these processes have not been described to date.

Thus, from emerging bioinformatics information, the concept arises that most HLA-A and HLA-B haplotypes are biased to present epitopes derived from TMHs. Our present knowledge of antigen presentation is exclusively based on that of soluble antigens, and we currently cannot explain how antigen presenting cells can self- and cross-present integral membrane proteins. Both these processes can be expected to require unique, yet unknown, mechanisms and this might well relate to the disease-associations of specific HLA haplotypes. For instance, members of the HLA-B27 super type are associated with spondyloarthropathies (psoriasis, inflammatory bowel disease, reactive arthritis, ankylosing spondylitis) (44). As we explained in this Opinion article, this super type preferentially presents epitopes that are immediately adjacent to transmembrane helices and perhaps medullary thymic epithelial cells cannot efficiently present these peptide fragments. This would result in incomplete negative selection and a population of self-reactive cytolytic T cells, which might contribute to the pathology of these inflammatory diseases. Evidence for such an incomplete self-tolerance of membrane-buried epitopes comes from the finding that a large proportion of healthy HLA-A02 restricted individuals possess naive cytolytic T cells specific for the immunodominant epitope from the TMH of Mart1 (45). Resolving how TMHs are processed for antigen self-presentation and cross-presentation should allow for a better understanding of immunology, and it might ultimately allow for new insights into the mechanisms of the disease-associations of HLA haplotypes.

## Methods

### Input Data

The UniProt human reference proteome UP000005640_9606 was used for all analyses.

### *In Silico* Predictions

Transmembrane helices for all human proteins were predicted using the TMHMM Server 2.0^1^ (23). HLA-binding 9-mers were predicted using a custom R implementation^2^ of the SMMPMBEC method (46). For each HLA super type, IC50 values for all 9-mers from the human proteome were predicted. The 2% peptides with the lowest IC50 values were defined as binders.

### Overlap Analysis

Of all 9-mers in the human proteome, 5.3% are predicted to overlap with TMHs (TMHs) by at least one amino acid (i.e., based on the sequence coverage of TMHs on the entire human proteome). Therefore, if there were no correlation between TMH overlap and HLA binding, the share of TMH peptides among predicted HLA binders would equally be 5.3%. As a statistical test of this null hypothesis, we used the binomial test. Specifically, the null hypothesis of this binomial test is that every predicted HLA binder is independently chosen at random to be TMH overlapping with probability 0.053. Confidence intervals for repeated sampling under this null hypothesis were determined from the critical region of this binomial test, for which we chose an alpha value of 0.001 (R code provided as Supplemental Information). Note that the independence assumption of the binomial test is approximate in this case, but because the predicted HLA binders constitute only 2% of the 9-mers in the human proteome, this approximation is reasonable.

### Hydrophobicity Index

Kyte–Doolittle scores were used to describe hydrophobicity of amino acids (22). The hydrophobicity of a peptide was calculated as the mean hydrophobicity of its constituting amino acids. Hydrophobicity indices for each HLA allele were calculated by multiplying the height of each amino acid in the binding motif with the Kyte–Doolittle score of the corresponding amino acid and averaging these values for all combinations of positions and amino acids. The mean hydrophobicity index of peptide in the human proteome was then subtracted for normalization. Binding motifs for this purpose were derived from the SMMPMBEC matrix M as explained in the Supporting Information.

### Control Set of Hydrophobic Epitopes from Soluble Protein Regions

To construct a control set of peptides derived from soluble protein regions (i.e., non-TMHs) with a similar hydrophobicity distribution as the peptides overlapping with TMHs, we determined each percentile of the TMH peptide hydrophobicity distribution and sampled equal amounts of soluble protein peptides within each percentile. Since by far most hydrophobic peptides overlap with TMHs (Figure 1C), we could only obtain a control set with a size 1/10 of the TMH peptide set.

### Reproducibility

The R scripts used to perform the analyses shown in this paper are available as Supplemental Material.

## Author Contributions

FB, JT, and GB conceived and designed the research; JT performed the bioinformatics; FB, JT, and GB analyzed the data. FB and GB wrote the manuscript; all authors read and approved the final manuscript.

## Conflict of Interest Statement

The authors declare that the research was conducted in the absence of any commercial or financial relationships that could be construed as a potential conflict of interest.

## Footnotes

^1^http://www.cbs.dtu.dk/services/TMHMM/.

^2^https://github.com/jtextor/epitope-prediction.
