# Supplementary material for: Transmembrane Helices Are an Overlooked Source of Major Histocompatibility Complex Class I Epitopes
Source: Front Immunol. 2017 Sep 11;8:1118. doi: 10.3389/fimmu.2017.01118 (PMC5604083; doi:10.3389/fimmu.2017.01118)
Supplement: Supplementary file 1 [file data_sheet_1.pdf]

Supplementary Information

Table S1

p-values for the null hypothesis that there is no statistical relationship between the distribution of predicted HLA binders and the distribution of predicted transmembrane helices, based on a binomial test (Methods).

HLA allele	p-value
A01-01	$< 10^{-20}$
A02-01	$< 10^{-20}$
A03-01	$< 10^{-20}$
A24-02	$< 10^{-20}$
A26-01	$< 10^{-20}$
B07-02	0.00015
B08-01	$< 10^{-20}$
B15-01	$< 10^{-20}$
B18-01	$3.04 \cdot 10^{-19}$
B27-05	$6.81 \cdot 10^{-18}$
B39-01	$< 10^{-20}$
B40-02	$< 10^{-20}$
B58-01	$< 10^{-20}$

Deriving Binding Motifs from SMMPMBEC Matrices

Let $M_{i,j}$ denote the matrix entry for amino acid j at position i. Each entry can be interpreted as the log contribution of an occurrence of the corresponding letter at the corresponding position to the total IC_{50} value; see the SMMPMBEC paper [1] for details.

Now, define another matrix $p_{i,j}$ as

$$p_{i,j} = \frac{10^{M_{i,j}}}{\sum_j 10^{M_{i,j}}} ,$$

that is, the columns of p are proportional to the IC_{50} values represented by M and each column sums up to 1. We compute the column-wise information content as

$$I_i = \log_{20} - \sum_j p_{i,j} \log p_{i,j} ,$$

where we use the logarithm of base e and define $0 \log(0) = 0$. The height of each letter in the binding motif is then given by

$$h_{i,j} = p_{i,j} I_i .$$

References

[1] Y. Kim, J. Sidney, C. Pinilla, A. Sette, and B. Peters. Derivation of an amino acid similarity matrix for peptide: MHC binding and its application as a Bayesian prior. *BMC Bioinformatics*, 10:394, 2009.
